# Power to heart rate ratio as a metric to optimize altitude training in professional cyclists

**DOI:** 10.5114/biolsport.2026.162049

**Published:** 2026-07-27

**Authors:** Basile Moreillon, Bastien Krumm, Lena Mettraux, Julian Wackernell, James Spragg, Martin Faulhaber, Raphael Faiss

**Affiliations:** 1 Research and Expertise in anti-Doping Sciences (REDs), Institute of Sport Sciences, University of Lausanne, Lausanne, Switzerland; 2 Swiss Federal Institute of Sport (SFISM), Magglingen, Switzerland; 3 Department of Sport Science, University of Innsbruck, Innsbruck, Austria; 4 Tudor Pro Cycling Team, Schenkon, Switzerland

**Keywords:** Altitude, Performance, Cycling, Training, Acclimatization, Hemoglobin mass

## Abstract

Hematological and physiological variations were assessed during an altitude training camp in professional cyclists, with precise training load monitoring. External to internal training load ratio defined as power output (W) per heartbeat (bpm) (W/HR) was investigated as a novel metric pertaining altitude acclimatization and training camp response. Nineteen male professional cyclists participated in a 3-week Live-High Train-Low (LHTL) camp. Blood samples were collected to assess hemoglobin concentration ([Hb]), hematocrit (HCT), reticulocyte percentage (RET%), and immature reticulocyte fraction (IRF). Total hemoglobin mass (Hbmass) and plasma volume (PV) were measured upon arrival (pre-) and on the last day (post-) at altitude. Training load was monitored across the camp with power output (W), duration (min), heart rate (HR, bpm), and total work (W; kJ) recorded. W/HR was computed from each session’s average W and HR. From Pre- to Post-, Hbmass increased by 3.7% (p < 0.001), while PV decreased by 4.4% (p < 0.05). [Hb], HCT, RET%, and IRF were elevated (+4.1%; +6.2%; +31.0%; and +50.0%; respectively). Training load increased progressively from the lead-in phase to the second week (+68%; +83%; respectively). W/HR decreased by 11% (p < 0.01) at the onset of the hypoxic exposure before improving gradually again. W/HR in normoxia was 7% higher post-camp compared to the lead in phase (p < 0.01). Overall, the altitude training elicited positive hematological adaptations in professional cyclists. The increase in W/HR after the camp could suggest an enhanced cardiovascular efficiency and/or peripheral oxygen utilization. W/HR may represent a pertinent metric beyond Hbmass with its concomitant response to altitude and training stimuli.

## INTRODUCTION

Athletes preparing for near-sea level competitions frequently perform training camps at altitude to target improved aerobic performance mostly through an increased total hemoglobin mass (Hbmass) in response to erythropoiesis triggered by the hypoxic environment.

Increased Hbmass may support an improved maximal oxygen consumption (${\dot{\text{V}}\text{O}}_{2\max}$) [1, 2] as a proxy of aerobic capacity in cycling [3]. However, the sole increase in Hbmass does not necessarily translate into aerobic performance improvement [4], underlining the necessity of adequate training in athletes to potentiate the increased arterial oxygen convection through enhanced diffusion at a peripheral level (i.e. higher capillary and mitochondrial density) [5–7].

However, the efficacy of altitude training strategies remains debated [8, 9], partly due to substantial interindividual variability in hematological responses, disrupted recovery or potential maladaptations [10–13]. While the hypoxic dose and the timing of return to sea-level before competition have been examined to optimize performance [14, 15], individual physiological responses remain paramount to understand if altitude-induced adaptations translate into improved athletic performance [16]. Several physiological mechanisms may actually underpin a lack of response or even negative outcomes observed in nearly half of the studies with prolonged altitude exposure [13, 17].

First, iron supplementation was reported as central if ferritin levels are lower than 130 mg/mL prior to altitude exposure to allow for an erythropoietic response [18, 19], even if this paradigm has recently been challenged [20].

Additionally, hypoxia induces challenges to fluid balance [21]: during the first days of altitude exposure, ventilatory rate increases as a response to lower blood oxygen saturation (SpO_2_), which induces greater respiratory fluid loss [11], and a down-regulation of the renin-angiotensin-aldosterone system [22]. This outlines the importance to control for hydration status in the context of an altitude training camp and the need to implement strategies to prevent dehydration [18, 23] to limit the [Hb] increase and optimize EPO secretion.

Training volume and intensity may be carefully prescribed to allow for training adaptations on top of the hypoxic stress. A short tapering period (i.e. a progressive reduction of the training load) [24, 25] is recommended before ascending to altitude. Following arrival, an acclimatization phase of 3–5 days should be implemented, during which training volume is reduced by approximately 25% relative to sea level, and high-intensity sessions are avoided [26]. This acclimatization phase is crucial to avoid any initial accumulation of fatigue due to strong catabolic and inflammatory responses reported during the first days of altitude exposure [27–29]. Training volume and intensity can subsequently be increased to usual levels [26, 30]. Mainly, training prescription is adapted upon the athlete’s day-today recovery to optimize the balance between hypoxic and training stress [30].

In that context, the distinction between external and internal training load can be considered when prescribing training [31]. External load can be characterized as the quantity and quality of training whereas internal load is defined by the psychophysiological response elicited for a given external training load [32]. For instance, monitoring heart rate (HR) (i.e. as an indicator reflecting internal training load) in relation to power output (W, marker of external load) could prove valuable to adjust training prescription in consideration of environmental stressors. In hypoxic conditions, HR will be elevated for a given power compared to sea-level [33], outlining a significant impact on internal load and the need to adapt external training load prescription. In other words, a given oxygen consumption will be associated with an absolute power output, which, in hypoxic conditions is achieved through increased ventilation, HR and diffusion at the muscle level [34].

Overall, monitoring W/HR may represent a relevant indicator of the altitude acclimatization [35]. Since athletes eventually target improved aerobic performance, and because there are logistical and regulation constraints to measure Hbmass at multiple timepoint, we suggest that W/HR may reflect positive outcomes from an altitude training camp as a result of a well-monitored training stimulus and the concomitant hypoxic exposure associated with increased erythropoiesis.

This study therefore aimed to investigate W/HR as a variable sensitive to physiological responses to a 3-weeks altitude training camp at 2020 m by investigating Hbmass and PV in 19 professional cyclists. W/HR measured in normoxia was hypothesized to be increased after the altitude camp in conjunction with an increased Hbmass.

## MATERIALS AND METHODS

### Study design

Nineteen (19) elite cyclists participated in a 18 (n = 6) or 21 days (n = 13) altitude camp at 2020 m (average temperature 16.6°C, daily high temperature 22.8°C, precipitation 0 mm, inspired oxygen pressure 114±3 mmHg, Kühtai, Austria) with a “Live High-Train Low” paradigm (LHTL), with “low” training performed < 600 m (Inn Valley) [36, 37]. Blood samples were collected at 4 time-points: 1) 14 days before altitude exposure, 2) on the first day of altitude exposure, 3) on the last day of altitude exposure and 4) 3 days after return to sea level and, 5) 10 days after the return to sea level. The hypoxic dose was estimated in km · h^−1^ (with altitude in km (i.e. 2.05) multiplied by the time exposed at altitude as defined in [38]) and in % saturation hours (defined in [39] as (98/SpO_2_ – 1)·h·100, where SpO_2_ is the average morning peripheral oxygen saturation value (in %) and h the time (in hours). Additionally, circulating blood volume, namely Hbmass and plasma volume (PV), were measured on the first (within 24 h) and last day of altitude exposure (*Table 1*). Finally, urine specific gravity (USG), blood oxygen saturation (SpO_2_), sleep quality and training metrics were monitored daily. The experimental study design is represented in *Figure 1*. This study was part of a larger project that also evaluated hematological, steroidal and endocrine adaptations to altitude training in cyclists with all hematological variables available [40] so that the current article focused essentially on responses to altitude related to training load.

TABLE 1Average duration (min), distance (km), elevation (m), total work (*W*; kJ), mean relative power (W/kg), mean heart rate (bpm) and external to internal load ratio (W/HR) of all training sessions performed by professional cyclists before (lead-in), during (microcycles 1–6), and after (post-camp) an altitude training camp at 2020 m asl. n = number of training sessions. Statistical significance of the time-effect is shown in the last column.

**Table:** 

	Lead-in (n = 78 ± 12)	Day 1	Day 2	Day 3	Day 4	Day 5	Day 6	Day 7	Day 8

		Microcycle 1 (n = 58 ± 7)				Microcyle 2 (n = 63 ± 6)			
Duration (min)	194 ± 64	186 ± 32	229 ± 17	270 ± 16	75 ± 26	223 ± 31	252 ± 25	413 ± 112	124 ± 97

		201 ± 54				277 ± 40			

Distance (km)	70.0 ± 37	96.5 ± 9.4	97.1 ± 3.7	127 ± 9.7	35.5 ± 13.6	93.2 ± 14.0	109.9 ± 10.7	150.8 ± 11.0	39.4 ± 12.6

		96.7 ± 29.6				106.3 ± 38.0			

Elevation (m)	696 ± 509	831 ± 178	1700 ± 429	1616 ± 142	227 ± 180	2129 ± 234	2020 ± 561	3062 ± 574	493 ± 929

		1203 ± 607				2117 ± 992			

Total Work (kJ)	1733 ± 827	1626 ± 202	2208 ± 223	2689 ± 304	573 ± 150	2941 ± 435	2728 ± 513	4030 ± 526	500 ± 205

		1921 ± 750				2860 ± 1177			

Mean Relative Power (W/kg)	2.8 ± 0.4	2.3 ± 0.2	2.7 ± 0.2	2.7 ± 0.2	2.2 ± 0.1	3.3 ± 0.3	2.9 ± 0.3	2.9 ± 0.2	2.0 ± 0.3

		2.5 ± 0.3				2.9 ± 0.5			

Mean Heart Rate (bpm)	126 ± 15	120 ± 10	131 ± 6	131 ± 8	119 ± 8	140 ± 8	136 ± 8	133 ± 7	107 ± 7

		126 ± 10				132 ± 13			

External to Internal Load Ratio (W/HR)	1.48 ± 0.17	1.30 ± 0.15	1.39 ± 0.11	1.41 ± 0.14	1.35 ± 0.16	1.60 ± 0.16	1.42 ± 0.15	1.51 ± 0.12	1.27 ± 0.17

		1.32 ± 0.15				1.45 ± 0.18

**Table:** 

	Day 9	Day 10	Day 11	Day 12	Day 13	Day 14	Day 15

	Microcycle 3 (n = 60 ± 5)				Microcycle 4 (n = 42 ± 4)		
Duration (min)	290 ± 27	188 ± 53	408 ± 40	58 ± 31	277 ± 63	406 ± 24	110 ± 113

	277 ± 34				309 ± 71		

Distance (km)	121.8 ± 10.1	79.1 ± 12.2	174.7 ± 31.8	31.0 ± 10.5	118.5 ± 20.5	180.6 ± 14.0	56.0 ± 56.1

	116.7 ± 51.9				138.7 ± 48.5		

Elevation (m)	2666 ± 250	1858 ± 872	3006 ± 663	238 ± 171	2299 ± 1268	3218 ± 1079	1569 ± 1195

	2302 ± 1039				2662 ± 1273		

Total Work (kJ)	3360 ± 285	2202 ± 911	4387 ± 673	488 ± 201	2795 ± 690	4398 ± 570	1319 ± 1272

	3007 ± 1381				3172 ± 1472		

Mean Relative Power (W/kg)	3.1 ± 0.2	3.0 ± 0.7	3.0 ± 0.2	2.1 ± 0.5	2.8 ± 0.3	2.9 ± 0.2	2.4 ± 0.5

	2.9 ± 0.5				2.7 ± 0.4		

Mean Heart Rate (bpm)	137 ± 7	129 ± 16	130 ± 7	110 ± 10	137 ± 8	125 ± 11	111 ± 15

	131 ± 12				128 ± 14		

External to Internal Load Ratio (W/HR)	1.54 ± 0.12	1.60 ± 0.16	1.58 ± 0.12	1.38 ± 0.09	1.37 ± 0.12	1.55 ± 0.10	1.47 ± 0.23

	1.51 ± 0.14				1.41 ± 0.15

**Table:** 

	Day 16	Day 17	Day 18	Day 19	Day 20	Post-camp (n = 86 ± 14)	Time-effect p-value

	Microcycle 5 (n = 39 ± 3)			Microcycle 6 (n = 17 ± 1)			
Duration (min)	242 ± 63	335 ± 68	66 ± 6	202 ± 89	254 ± 80	191 ± 65	N/a
	
	262 ± 60			263 ± 56			< 0.001

Distance (km)	93.2 ± 27.9	125.4 ± 13.0	24.6 ± 5.3	122.7 ± 15.7	121.4 ± 32.4	117.0 ± 61	N/a
	
	97.5 ± 37.9			122.3 ± 20.5			< 0.001

Elevation (m)	1837 ± 977	2652 ± 904	434 ± 94	1576 ± 748	951 ± 901	1374 ± 1045	N/a
	
	1978 ± 1128			1420 ± 807			< 0.001

Total Work (kJ)	2406 ± 869	3347 ± 681	569 ± 130	2559 ± 756	2608 ± 1285	2513 ± 1332	N/a
	
	2553 ± 1141			2572 ± 890			< 0.001

Mean Relative Power (W/kg)	2.8 ± 0.4	2.8 ± 0.2	2.3 ± 0.3	2.7 ± 0.3	2.7 ± 0.3	3.2 ± 0.6	N/a
	
	2.8 ± 0.4			2.7 ± 0.3			< 0.001

Mean Heart Rate (bpm)	131 ± 13	127 ± 6.6	108 ± 11	133 ± 6	129 ± 8	134 ± 13	N/a
	
	127 ± 13			131 ± 7			< 0.001

External to Internal Load Ratio (W/HR)	1.45 ± 0.11	1.52 ± 0.09	1.44 ± 0.12	1.46 ± 0.16	1.55 ± 0.17	1.58 ± 0.21	N/a
	
	1.43 ± 0.10			1.36 ± 0.14			< 0.001

**FIG. 1:**
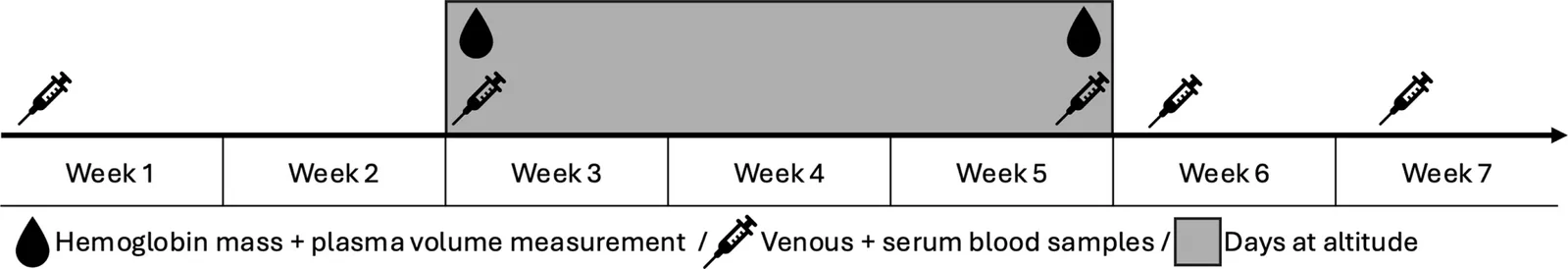
Experimental study design

### Study subjects

All subjects were elite male professional cyclists (24.6 ± 2.7 y. o., 68.3 ± 6.1 kg, maximal aerobic power (MAP) 560 ± 34 W) [41], competing at the Union Cycliste Internationale (UCI) World Tour & Continental Pro level. Participants voluntarily took part in a training camp organized by their professional cycling team and resided at an altitude of 2020 m asl. (inspired oxygen pressure 114 ± 3 mmHg) for 18 (n = 6) to 21 days (n = 13). Procedures and risks were fully explained to the participants, and all provided their written consent to participate in the study. This study was approved by both local ethics committees (CER-VD, Lausanne, Switzerland, Agreement 2020-00549; Board for Ethical Questions in Science of the University of Innsbruck, Agreement 50/2024) and conducted in accordance with the Declaration of Helsinki.

### Blood sampling

Blood sampling was performed by trained phlebotomists under supervision of the medical staff of the cycling team, and following the very strict and standardized blood collection guidelines from the World Anti-Doping Agency (WADA) in order to minimize any influence of pre-analytical confounders [42, 43]. The participants reported to the lab having avoided any physical exercise in the 2 hours preceding the sampling. The subjects then remained seated for 10 min to stabilize their PV [44]. Both whole blood (3 mL; BD Vacutainer^®^ K2EDTA tubes, Becton Dickinson, New Jersey, USA) and serum (5 mL; BD Vacutainer^®^ SSTTM II serum separator Advance tubes Becton Dickinson, New Jersey, USA) samples were collected. Whole blood samples were immediately homogenized, and all tubes (whole blood and serum samples) were then stored at 4° C before being transported the same day to Lausanne in a refrigerated container. Whole blood samples were subsequently analyzed in duplicate by flow cytometry (Sysmex XN-1000, Sysmex, Norderstedt, Germany). The following variables were recorded: [Hb], Hematocrit (HCT), Reticulocytes percentage (RET%) and Immature Reticulocyte Fraction (IRF).

Upon reception at the laboratory, serum tubes were centrifuged, and serum aliquots were frozen at -80°C for later serological analyses in a single batch. Concentrations of ferritin were determined using a dedicated automated clinical chemistry system (Biolis 30i, Boeki Medisys Inc., Tokyo, Japan). All analyses were performed on the same instrument following adequate calibration as recommended by the manufacturer with all samples for a given participant analyzed in the same batch.

### Total hemoglobin mass and plasma volume measurements

A fully automated system was used to determine total red blood cell volume (RBCV), PV and Hbmass using a carbon monoxide (CO) rebreathing procedure (OpCo: Detalo Instruments, Birkerod, Denmark), which is outlined in detail elsewhere [45]. The procedure was performed in duplicate to limit analytical variability. The typical total error for the determination of Hbmass using this procedure in our laboratory is < 1.5%, in line with existing studies [45].

### Individual training monitoring and prescription

Training sessions were prescribed individually by the team coaches with 6 microcycles separated by recovery days over the altitude stay, respectively named M1, M2, …, M6. Additionally, data from training sessions performed during the week preceding the start of the camp were grouped into a lead-in phase, and, similarly, sessions performed until 1 week after the end of the camp were grouped into a post-camp phase. During each individual training session, the following variables were measured using the subjects’ powermeter (RED AXS, Sram LLX, Chicago, USA; athletes performed a 0 calibration at the onset of each training session) and bike computer (Elemnt Ace GPS bike computer, Wahoo Fitness, Atlanta, USA): total duration (min), total distance (km), total work (*W*; kJ), power (W; sampling rate = 1 Hz), heart rate (HR; BPM; TRACKR Heart Rate Monitor Chest Strap, Wahoo Fitness, Atlanta, USA) and cumulated elevation gains (m).

To consider the role of the initial hypoxic ventilatory response causing an increase in heart rate at altitude followed by subsequent progressive acclimatization [46], the internal load response to cycling was assessed by calculating the external to internal training load ratio (W/HR). Average W/HR was calculated from every on-bike activity available (all training sessions and races) performed between 1 week prior to the start of the camp to 1 week after the end of the camp. W/HR was computed taking each session’s mean W and HR to minimize the impact of the HR delay in response to sharp increase in exercise intensity. W/HR was considered a variable reflecting the progressive adaptation to altitude in cyclists as an increase in this metric can be characterized by an increase in *W* for a given HR.

Additionally, time in different training intensity zones was monitored and zones were defined as percentage of HRmax. The first intensity zone (Z1) was defined as any intensity below 77% of HRmax, the second (Z2) as intensities between 77% and 87% and the third (Z3) as anything above 87% [47, 48].

### Daily monitoring of heart rate, oxygen saturation, resting heart rate

Subjects were asked each morning to measure their resting heart rate (RHR) and SpO_2_ (Onyx II 9550, Nonin Medical Inc, Plymouth, USA) for which 1-min average values were recorded, and to report sleep quality on a visual analogic scale (VAS, 1–10) which included the following items: a) *how many hours did you sleep?*, b) *how well did you sleep?* (1 = terrible, 10 = perfectly), *how fresh do you feel this morning?* (1 = very tired, 10 = very fresh).

### Hydration and Nutrition guidelines

Each morning, subjects weighed themselves and filled a cup with urine from which specific gravity (USG) was measured (KERN ORAP Refraktometer, Kern & Sohn Gmbh, Balingen, Deutschland). USG higher than 1.020 was considered as an indication of dehydration and athletes were hence recommended to adjust their daily hydration [23].

Carbohydrate intake recommendations were adjusted between 8–12 g/kg/day for all athletes [49] depending on training content.

Iron availability was determined prior to the study with ferritin concentration being analyzed through serum blood sample 6 weeks before the altitude training camp. Supplemental iron was prescribed in accordance with the team doctor with 100 mg of oral elemental iron (Tardyferon^®^, ferrous sulfate) supplemented every other day for 4 weeks prior to altitude if ferritin levels were < 100 ng/mL. Iron supplementation was provided if required following current recommendations based on iron availability during altitude exposure.

### Statistical analyses

Data are presented as means, standard deviations (± SD) and/or 95% confidence intervals (CI: [5:95]). Normality of the distributions was assessed by the D’Agostino & Pearson test. Residual plots were visually inspected to exclude any obvious deviation from homoscedasticity. The effect of altitude exposure on the blood variables ([Hb], RET%, HCT and IRF) were analysed using a multivariate linear mixed-effects model with multiple comparisons to determine whether changes in dependent (i.e. physiological variables) variables differed over time (fixed factor). Total duration (min), total distance (km), *W* (kJ), mean relative power (W/kg), mean heart rate (bpm), elevation (m) and W/HR ratio data were grouped into microcycles, as mentioned above in the *study design* section, and two-way ANOVAs with multiple comparisons and microcycles and subjects as factors were performed. Finally, differences between pre and post values for Hbmass and PV were tested with a paired T-test. Statistical association between change in Hbmass and meaaured variables was assessed by determining Pearson’s correlation coefficients. Statistical analyses and graphs were computed using a dedicated GraphPad Prism 10 software (GraphPad Software, LLC, Boston, USA). The null hypothesis was rejected for p < 0.05.

## RESULTS

### Hypoxic dose

The hypoxic dose represented ~775 km · h^−1^ after 18 days and ~900 km·h^−1^ after 21 days. The individual hypoxic dose represented 1387±524 saturation hours.

### Hematological variables

Hbmass significantly increased by 3.7 ± 3.5% after 18 to 21 days at 2020 m a.s.l. (1008 ± 118 g) compared to baseline (972 ± 123 g; (95% CI: [19, 52] g, p < 0.001)). Conversely, a significant PV decrease of 230 ± 266 mL (95% CI: [-102, -359] ml, p < 0.01; -4.4%) was noted after altitude exposure (3540 ± 432 mL) compared to the start of altitude (3770 ± 454 mL). There was no correlation observed between the individual changes in Hbmass and any of the variables recorded. Individual Hbmass and PV variables are presented graphically in *Figure 2*.

**FIG. 2:**
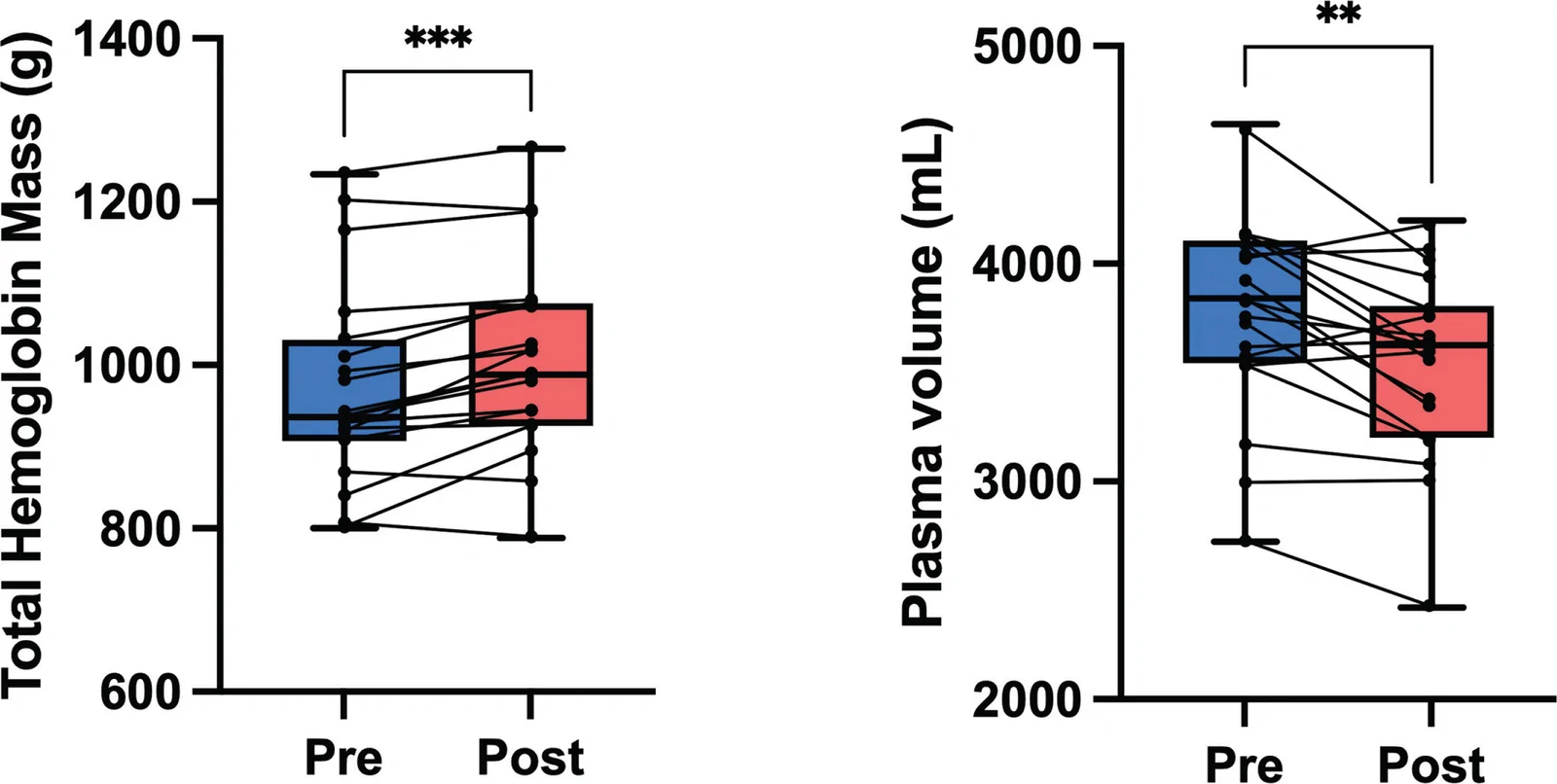
Total hemoglobin mass (g) and plasma volume (mL) on the first day of altitude exposure (Pre) compared to the last day of altitude exposure (Post). Boxplots present median, first and third quartiles, maximum and minimum values. *** p<0.001 and ** p<0.01 for differences between timepoints.

After 21 days at altitude, [Hb] was increased from 14.6 ± 1.1 g×dL^−1^ to 15.3 ± 1.0 g×dL^−1^ (95% CI: [+0.4, +1.0] g×dL^−1^, p < 0.01). RET%, HCT and IRF all significantly increased, respectively from 1.10 ± 0.30% to 1.46 ± 0.32% (95% CI: [+0.24, +0.49], p < 0.001), from 43.3 ± 3.1% to 46.3 ± 2.5% (95% CI: [+2.0, +3.9]. p < 0.001), and from 4.3 ± 1.2% to 6.5 ± 3.0% (95% CI: [+0.9, +3.4], p < 0.01).

Ferritin levels were 92 ± 37 (95% CI: [+75, +109]) at baseline and remained stable with no significant changes over the monitored period (coefficient of variation = 3.6%).

### External to internal load ratio

A significant decrease of W/HR was observed between the lead-in and the first 4 days (M1) (-0.11 ± 0.03, p < 0.001), which then progressively increased until the 12^th^ day (M2, +0.11 ± 0.03, p < 0.001; M3, +0.19 ± 0.03, p < 0.001; respectively). This increase was followed by a significantly lower value in the following microcycle (M4) (-0.11 ± 0.03, p < 0.01). When comparing lead-in to post-camp values a significant increase was noted (+0.09 ± 0.02, CI: [0.01, 0.15], p < 0.01). All values are presented in *Table 1* and *Figure 3*.

**FIG. 3:**
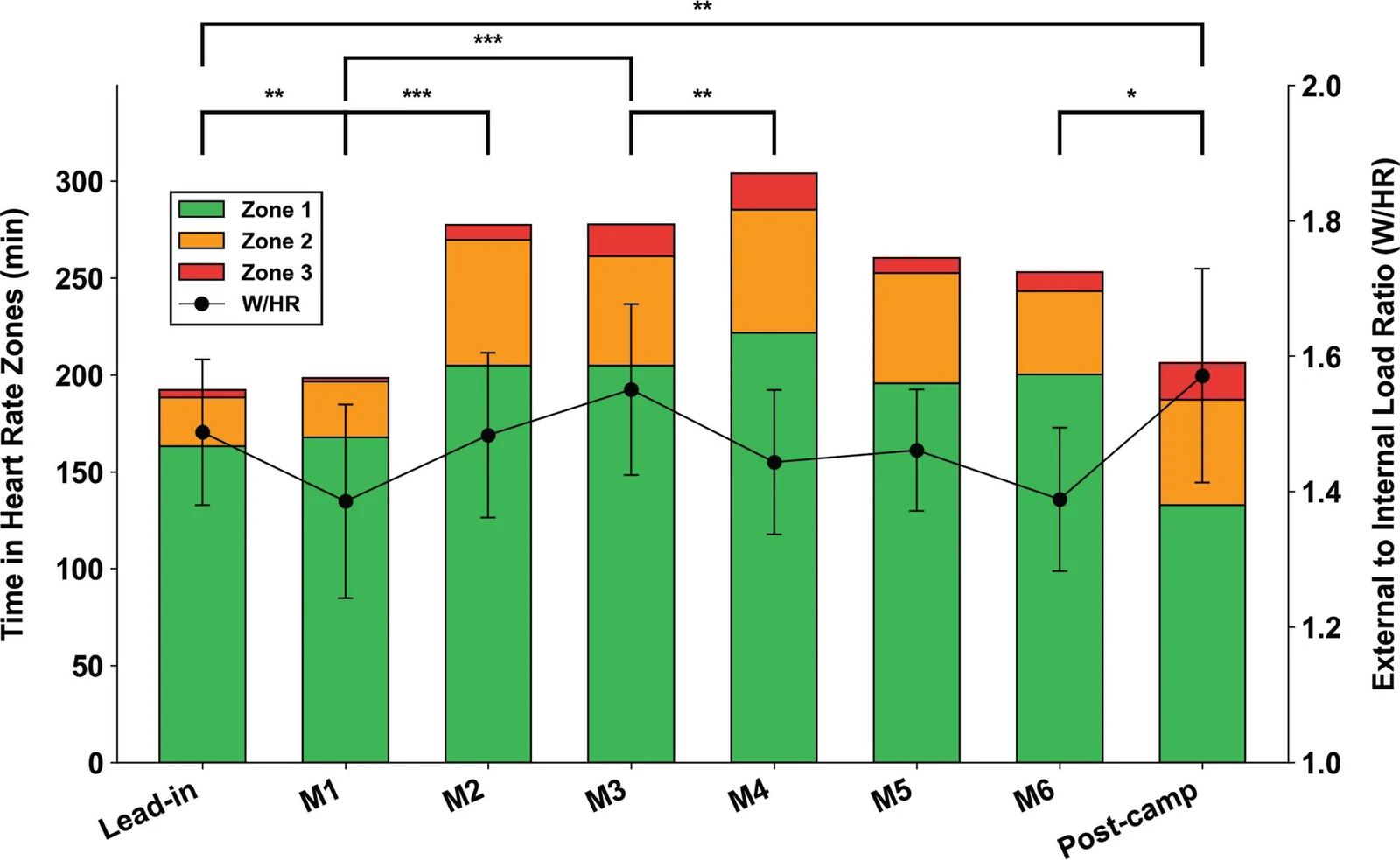
Training time spent in heart rate (HR) zones per microcycle before (lead-in), during and after (post-camp) an altitude training camp at 2020m asl. HR zone 1 (green, Z1) is defined as intensities below 77% of maximum HR, HR zone 2 (orange, Z2) between 77% to 87% and HR zone 3 (red, Z3) as above 87% (left Y axcis), and external to internal load ratio (W/HR, mean ± SD, right Y axis) per microcycle before (lead-in), during and after (post-camp) an altitude training camp at 2020m asl. ***, p<0.001, ** p<0.01 and * p<0.05 for differences between timepoints for W/HR.

### Training load monitoring

Variables illustrating training load (duration (min), distance (km), elevation (m), *W* (kJ), mean relative power (W/kg) and mean HR (bpm)), are presented in *Table 1* where the 6 microcycles can be visually identified. Rest or recovery days were scheduled on day 4, 8, 12, 15, and 18 in a majority of athletes and generally comprised either a very short bicycle ride or no exercise at all. The individual training load prescription led to a larger dispersion of training variables in days 15 and 20 notably for duration (110 ± 113 min; 254 ± 80 min; respectively), *W* (1319 ± 1272 kJ; 2608 ± 1285 kJ; respectively), distance (56.0 ± 56.1 km; 121.4 ± 32.4 km; respectively) and elevation (1319 ± 1195 m; 951 ± 901 m; respectively).

Training load increased progressively from the lead-in week to the second week at altitude (M4) with higher training duration (+105 ± 22 min, p < 0.001), distance (+72 ± 8 km, p < 0.001), elevation (+1928 ± 181 m, p < 0.001) and *W* (+1415 ± 217 kJ, p < 0.001), with M4 being the microcycle with the highest external training load recorded. For those variables, all comparisons between microcycles from the first 2 weeks of the training camp presented a significant increase (p < 0.01 for all comparisons), except for the difference in duration and *W* between the lead-in and the first 4 days. A significant decrease after the end of the second week (between M4 and M5) was also noted for distance and elevation (-41.8 ± 9.8 km, p < 0.001; -683 ± 212 m, p < 0.05, respectively), but not for duration (-44 ± 24 min, p = 0.6) nor *W* (-623 kJ, p = 0.20). Similarly, duration, elevation and *W* were significantly lower between the end of the second week (M4) and the post-camp (-100 ± 21 min, p < 0.001; -1282 ± 179 m, p < 0.001; -642 ± 211 kJ, p < 0.05; respectively). Conversely, mean relative power significantly decreased between the lead-in and the first 4 days (M1) (-0.3 ± 0.1 W/kg, p < 0.01) but then significantly increased in the following 8 days (M2 and M3) (+0.4 ± 0.1 W/kg, p < 0.001; +0.4 ± 0.1 W/kg, p < 0.001; respectively). Post-camp mean relative power (2.8 ± 0.3 W/kg) was significantly elevated compared to all previous timepoints (p < 0.05 for all comparisons). On the other hand, mean HR (bpm) was only significantly elevated at the post-camp compared to lead-in, the first (M1) and the last microcycle (M5) (-7 ± 2 bpm, p < 0.01; -8 ± 2 bpm, p < 0.01; -8 ± 2 bpm, p < 0.05; respectively). All values are presented in *Table 1*.

Training intensity is illustrated with the time in the defined intensity zones in Figure *4*.

**FIG. 4:**
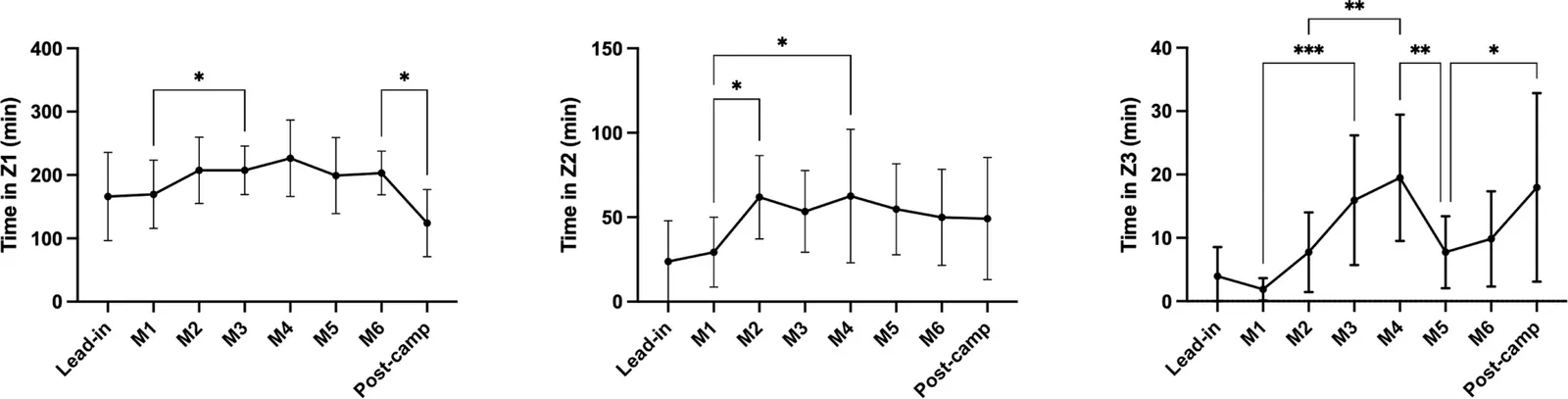
Training time spent in heart rate (HR) zones per microcycles before (lead-in), during and after (post-camp) an altitude training camp at 2020m asl. HR zone 1 (Z1) is defined as intensities below 77% of maximum HR, HR zone 2 (Z2) between 77% to 87% and HR zone 3 (Z3) as above 87%. A: time spent in Z1 ± SD. B: time spent in Z2 ± SD. C: time spent in Z3 ± SD. ***, p<0.001, ** p<0.01 and * p<0.05 for differences between timepoints.

### Daily monitoring variables

Average USG was 1.017 ± 0.005 (n = 343) and 88 measures were above the threshold of 1.02 (26%) while consecutive measures above that threshold occurred on 17 occasions throughout the training camp. Mean sleeping hours were 7.8 ± 0.8 h (n = 324) and sleep quality averaged 6.5 ± 1.7 (n = 324), while freshness averaged 6.0 ± 1.8.

RHR values averaged 52 ± 7 bpm (95% CI: [49, 56]) (n = 329) and average morning SpO_2_ was 94.5 ± 1 (95% CI: [94, 95]).

## DISCUSSION

The present study investigated the physiological adaptations induced by a 3-weeks altitude camp in 19 professional cyclists in relation with training load management. The results suggest that external to internal load ratio monitoring may prove to be valuable to guide training load management in elite endurance athletes.

### Hematological adaptations and iron metabolism

As hypothesized, an increase in Hbmass was noted after the hypoxic exposure, a strong indicator of positive performance adaptations, while PV decreased (*Figure 2*), in line with results found earlier in cyclists [50, 51] and endurance athletes [2, 52]. Despite elevated markers of erythropoiesis, a downshift in PV explained the hemoconcentration observed at the end of the altitude camp with increased [Hb] and HCT. The contraction of plasma volume (PV) may result from a shift in water from intra- to extra-vascular compartments [53–55], emphasizing the importance to control hydration status during altitude exposure and implement hydration strategies. Additionally, ferritin levels remained stable over the monitored period.

### Training load monitoring

Concomitantly with Hbmass, post-camp W/HR was increased after the altitude training camp (*Figure 3*). This reflects an absolute progression of 11 W, from 176 W to 187 W, for a given HR of 120 bpm, which is an intensity at which elite cyclists spend a significant amount of time throughout a race [56]. The ability to produce more external power per heartbeat may have resulted from a combination of elevated oxygen convection (due to the increased Hbmass) and enhanced diffusion at the muscle level (due to adequate training during the camp). Oxygen consumption is defined as the combination of oxygen convection (dependent on the cardiac stroke volume and heart rate) and local diffusion (illustrated by the arterio-venous difference in oxygen content [57]. Any change in circulating blood volumes (e.g., lower PV in an acclimatization phase at altitude or higher red blood cel volume after an adequate stay at altitude) would affect the cardiac ejection fraction. HR at a given power output may hence represent a general physiological surrogate of the cardiac output (i.e. cardiac function). In the context of altitude training, W/HR should however be viewed as a non-specific composite indicator, only reflecting the interaction between external load, internal response, and environmental stress. In lack of association with hematological responses during the altitude sojourn (e.g., gain in Hbmass), W/HR should not be considered a direct marker of hypoxia-induced physiological adaptations, despite probably providing trainers and athletes an objective indicator to eventually adjust their training content.

Nevertheless, the elevated W/HR observed in our study could suggest an improved – or at least maintained- oxygen uptake at the tissue level despite HR possibly confounded by external factors such as heat, stress of fatigue [23, 58, 59] and the maintenance of the power output putatively influenced by drafting during group rides or environmental conditions (i.e. temperature and wind). Increased oxygen convection through elevated blood oxygen content has been well documented [2, 52], and in line with the elevated Hbmass in this study. On the other hand, while the arterio-venous difference in oxygen content was not assessed in this study, peripheral adaptation (i.e. improved capillarization or mitochondrial density) are normally potentiated through high intensity training [6, 7] as prescribed near sea-level to athletes in the present study with 30 to 60 min spent over 87% of HRmax in day 9 and 13 (*Table 1*). Overall, further studies may consider both improved Hbmass and W/HR as positive indicators of the effects of altitude training in elite cyclists.

In this study, W/HR immediately declined upon arrival at altitude, but progressively returned close to baseline values at the onset of the second week (M3) (*Figure 3*). The initial decline may be explained by an increased HR at exercise to compensate the diminished oxygen availability through an elevated cardiac output [60] and indicates that training load should be reduced to allow acclimatization. This is showed by a lower relative power (reflecting training intensity in W/kg) as well as exercise duration (*Table 1*) during the first days of altitude exposure.

This training prescription pattern is in accordance with the latest scientific recommendations for acclimatization to altitude [26, 30] as team coaches consciously waited for athletes to acclimatize and W/HR to get back to baseline values before prescribing very intense workouts. The pre-altitude decrease in training load, corresponded on average to 34% less training hours and 39% lower total training work, slightly above the 25% training volume reduction recommended in the literature [26]. It should however be acknowledged that the altitude training camp took place immediately after the week including national championships races for which most athletes would possibly already taper. Reducing training load during the first 3 days at altitude by ~30% may have contributed to an effective acclimatization to the hypoxic environment illustrated by the increase in the external to internal load ratio (i.e. W/HR) between the first and second week at altitude.

The return of W/HR to near baseline values during the second week of altitude sojourn (*Figure 3*) is accompanied by an increase in *W* (*Table 1*), total exercise time and time spent in Z3 (*Figure 4*) with the latter being associated with high intensity interval training which was performed most of the time at low altitude [47]. Training load was then maintained which caused a decreased W/HR towards the end of the sojourn. A further decrease in W/HR was associated with diminished *W*, exercise duration and time spent in Z3 to optimize recovery and race performance on return to sea-level. The possible ranges for power output (e.g. from 0 to > 1500 W in professional cyclists) practically explain the lowered W/HR during certain microcycles (*Figure 3*) of the training camp. Heart rate values hence reflected the much lesser amplitude range (from < 40 to > 200 beats per minute) compared to power output (*Table 1*).

In summary, W/HR may prove to be a useful indicator to prescribe training load during altitude training. In that context, it is recommended to prescribe lower training loads, mainly through diminished intensity, to athletes over the first days of altitude exposure until W/ HR returns to baseline values. Training intensity can then be increased which induce a decline of that metric towards the end of the camp.

### Practical considerations

Altitude training strategies are deemed relevant by athletes and trainers to enhance performance, particularly through hematological adaptations such as increased Hbmass.

Considering the cost and constraints related to altitude training camps, it is important to consider the individual variability in the responses to hypoxia and the adaptation to training at altitude. When best-practice recommendations are met (i.e. adequate hypoxic dose, initial ferritin levels, hydration, diet, recovery, etc. [18, 61]), it remains crucial to implement adequate training strategies to eventually observe relevant performance improvements. Monitoring adequate markers of the external load and individual responses (internal load) therefore appears paramount to dynamically adjust training to find the adequate balance between anabolic and catabolic responses specific to training at altitude [40].

The observational study design with professional cyclists did neither allow for a normoxic control group, nor for the assessment of direct performance outcomes (maximal oxygen uptake, time-trial performance or lactate threshold, for example).

In this context, W/HR should primarily be framed as a monitoring and decision-support tool, that does not necessarily constitute a validated proxy for performance enhancement with altitude training.

In practice, W/HR could be determined through standardized and controlled procedures by asking the athletes to produce a submaximal (Zone 1) individual effort in reproducible conditions (ideally with no wind, no drafting, no heavy on the same or a similar terrain). Power output and the corresponding hear rate may then be recorded over a sufficient duration (e.g., 5 or 10 min). The measurement may be done at the onset of exercise or at the end of the training to possibly assess the effect of residual fatigue or accumulated training work. As cyclists usually perform a large amount of training in Zone 1 (see Figure 3), W/HR data may also be retrieved in any training file *a posteriori*, as files may include a map from which an adequate interval (e.g., a slow-paced climb with less influence of drafting or wind) can be selected.

Accordingly, altitude training shall typically include careful monitoring and individual adjustment to account for these complexities and optimize outcomes, where W/HR could further be investigated in conjunction with robust performance indicators.

## CONCLUSIONS

In conclusion, 18 to 21 days of LHTL at an altitude of 2020 m combined with a carefully monitored training programme was reported here as an efficient strategy to elicit positive physiological adaptations for athletic performance in professional cyclists. Large inter-individual variations, in line with previous studies with athletes at altitude, outlined that personal training prescription and monitoring were paramount to allow for positive outcomes. The increase in the ratio between external and internal load (measured as W/HR) observed after the camp could suggest sustained cardiovascular and peripheral adaptations following altitude training camps. Because positive effects of altitude training camps for elite athletes are nevertheless still debated, future studies may further investigate W/HR as a promising objective metric of exercise response before, during and after the hypoxic exposure.

## Data Availability

Data is available on reasonable request to the corresponding author with due regards to the fact that only de-identified data was being stored after analysis for compliance with the ethical requirements.
